# Perspectives on the Designation of Oligonucleotide Starting Materials

**DOI:** 10.1089/nat.2020.0909

**Published:** 2021-03-25

**Authors:** William F. Kiesman, Andrew K. McPherson, Louis J. Diorazio, Leo Van den Bergh, Peter D. Smith, John M. Northall, Alec Fettes, Tiejun Wang, Martin Mehlmann, Syed Raza, Gary Held

**Affiliations:** ^1^ Antisense Oligonucleotide Development and Manufacturing, Biogen, Inc., Cambridge, Massachusetts, USA.; ^2^ Process Organic Chemistry, Ionis Pharmaceuticals, Inc., Carlsbad, California, USA.; ^3^ Chemical Development, Pharmaceutical Technology & Development, Operations, AstraZeneca, Macclesfield, United Kingdom.; ^4^ API Small Molecule Development, Janssen, Beerse, Belgium.; ^5^ Early Chemical Development, Pharmaceutical Sciences, R&D, AstraZeneca, Macclesfield, United Kingdom.; ^6^ Chemical Development, Product Development and Supply, GlaxoSmithKline, Stevenage, United Kingdom.; ^7^ Pharmaceutical Division, Small Molecule Technical Development, Department of Process Chemistry and Catalysis, F. Hoffmann-La Roche Ltd., Basel, Switzerland.; ^8^ Global Regulatory Affairs, CMC & Devices, Sanofi, Bridgewater, New Jersey, USA.; ^9^ External Technical Oversight Analytics, F. Hoffmann-La Roche Ltd., Basel, Switzerland.; ^10^ Amidite Manufacturing and Process Development, Thermo Fisher Scientific, Milwaukee, Wisconsin, USA.; ^11^ Amidite Quality Control and Analytical Development, Thermo Fisher Scientific, Milwaukee, Wisconsin, USA.

**Keywords:** starting materials, regulatory, EPOC

## Abstract

The designation of starting materials (SMs) for pharmaceuticals has been a topic of great interest and debate since the first ICH quality guidance was published. The increase in the number and variety of commercialized oligonucleotides (antisense oligonucleotides—ASOs, small interfering RNAs—siRNAs, etc.) in recent years has reignited dialogue on this topic because of the unique complexity of the monomeric nucleotides and other contributory materials used to manufacture oligonucleotides. The SM working group in the European Pharma Oligonucleotide Consortium (EPOC) was formed to help establish simple, risk-based criteria to guide the justification of oligonucleotide SMs. This article provides a description of the common types of SMs, classes of SM impurities, and control strategies that will be helpful to maintain manufacturing consistency.

## Introduction

A harmonized approach for the designation and justification of starting materials (SMs) for new chemical entities (NCEs) has been outlined in recent regulatory guidance [1,2] and proposals from industry groups [3]. These risk-based approaches provide insights into how SMs can impact drug substance quality and also mechanisms for control of critical attributes of SMs that may impact drug substance quality.

As chemically synthesized active ingredients, oligonucleotides have the potential to share similar risk-based justifications as more traditional, small molecule NCEs. This anticipation is hindered, however, by the lack of recognized standards and the small numbers of approved oligonucleotide products. Such a situation could lead to justification of SMs for oligonucleotide products being subject to inconsistent expectations by agencies in different regions or, indeed, by sponsor companies.

The European Pharma Oligonucleotide Consortium (EPOC) [4] was created in 2018 to address this and similar situations. EPOC is a collaboration between multiple pharma companies with the aim of sharing chemistry, manufacturing and control (CMC) knowledge, and strategies to enable harmonization of oligonucleotide development and commercialization practices. The consortium will publish science-based recommendations for the development of oligonucleotide therapeutics in a series of technical white papers. These draw on its collective subject matter expertise, complementing that in the literature and will serve as a reference for industry practice and to help establish development principles for oligonucleotides. The consortium aims to be proactive and inclusive, and anticipates initiating wider discussion on oligonucleotide CMC practice and policy to expedite access to potentially life-changing medicines.

Within EPOC, the Oligonucleotide SMs Working Group was launched to examine member company practices and propose risk-based strategies for more uniform oligonucleotide SM justification packages.

This article summarizes general approaches to the justification packages that include the following:

- Determination of the criticality of SM impurities
- Illustration using deoxy phosphoramidites with typical quality attributes of SMs and analytical methods used for controls
- Application of justification to more complex phosphoramidites, for example, 2′-(methoxyethoxy)ribose (MOE) and locked nucleic acids (LNAs)
- Extension to convergent syntheses of oligonucleotides from smaller oligonucleotides such as dimers/blockmers
- Approaches for components of conjugates (linkers and ligands)

A broad range of SMs has been applied to the manufacture of therapeutic oligonucleotides and it is not feasible to cover all of the options. Rather than provide hard and fast rules, this report illustrates principles to consider for simpler SMs and elaborates this as the perceived complexity increases. In this way, sponsor companies can adapt and apply these principles to justification and specification of oligonucleotide SMs in the context of their own drug projects and corporate approaches to regulatory filing.

Oligonucleotide therapeutics are becoming more prevalent in the global marketplace and manufacturing scales for these complex products are increasing as larger volume indications become legitimate targets. A flexible, harmonized risk-based SM justification approach shared by regulators and developers will help ensure sustainable patient access to affordable, high-quality products.

## Discussion

### The manufacturing process

Before discussing SMs, it is necessary to understand the oligonucleotide manufacturing process to provide a context for justification of certain materials as SMs. The range of operations that are conducted may act as purging steps for SM-derived impurities and must be considered in any justification.

**FIG. 1.**
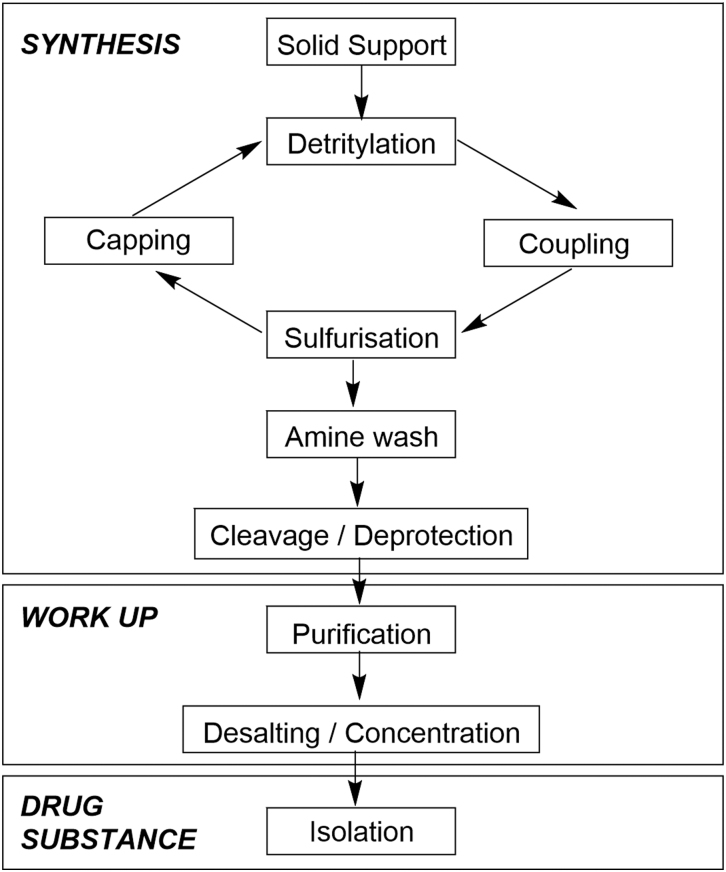
Process overview of oligonucleotide manufacturing operations.

As an end-to-end manufacturing process, the various operations can be grouped into three main activities that are conceptually similar to those employed in standard small-molecule preparation. These are Synthesis, Work-up (often referred to as Downstream Processing for oligonucleotides), and Drug Substance Isolation (Fig. 1).

**FIG. 2.**
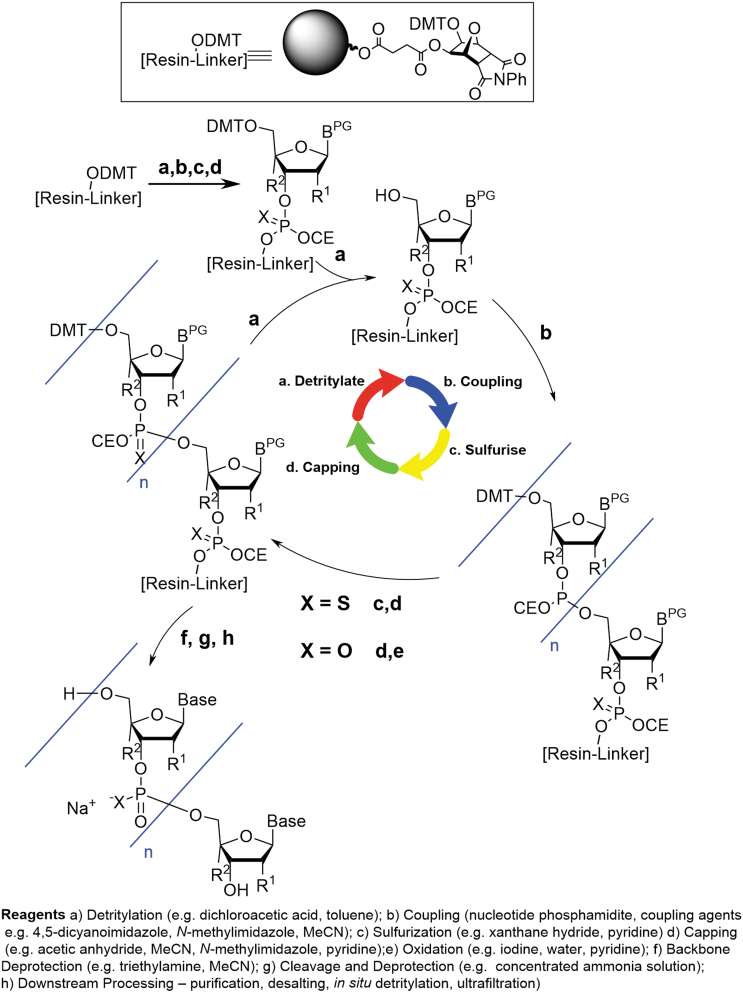
Typical oligonucleotide synthesis process.

When examined in more detail, however, the oligonucleotide process is different from small molecule manufacturing. The chemical synthesis of a therapeutic oligonucleotide is most often carried out on a functionalized solid support using an automated synthesizer. The oligonucleotide chain is extended through iterative synthetic cycles where each cycle results in the incorporation of one additional nucleotide unit (Fig. 2). The cycle consists of four successive steps:

- Detritylation: removal of a 4,4′-dimethoxytrityl (DMT) protecting group at the site where chain elongation will occur
- Coupling: reaction with an activated phosphoramidite-functionalized building block to enable introduction of a single-nucleotide unit into the growing oligonucleotide chain
- Sulfurization/Oxidation: introduction of a sulfur or oxygen atom at the newly created internucleotide phosphotriester linkage, resulting in conversion from P(III) to P(V)
- Capping: addition of a reactive acylating reagent to effect capping of any unreacted hydroxyl center remaining as a result of incomplete coupling or undesired deprotection side-reactions and reduce propagation of such impurities

Each step is highly selective and very high yielding and the solid support is thoroughly washed with solvent between each successive operation in the cycle. In combination with the high solubility of excess reagents and associated byproducts, this ensures that there is no carry-over of reagents, building blocks, and nontethered impurities between the different synthetic steps.*

The solid-supported synthesis is carried out as a single continuous operation. For a 20-mer oligonucleotide, this means a total of ∼80 synthetic steps carried out sequentially without pause in the process or isolated intermediates. The solid-supported synthesis is performed on a packed column with all reagents, solvents, and building blocks delivered as solutions under computer-programmed control. When combined with the advantages already mentioned (robust, high yielding, and highly selective chemistry with extensive column washing steps), this results in a highly controlled and predictable outcome for the synthesis phase.

Once the oligonucleotide sequence has been completed, deprotection steps are required before the work-up/downstream processing steps (purification, desalting, and concentration). The first operation is an amine wash to effect removal of the phosphorous backbone protecting group (typically 2-cyanoethyl, CE) and results in a global backbone deprotection of the phosphate/phosphorothioate esters, giving the triethylamine salt of the resin-bound oligonucleotide.

Treatment of the resin-supported oligonucleotide with aqueous ammonia removes various amine protecting groups on the nucleobases, as well as triggering cleavage of the resin linker, resulting in release of the oligonucleotide from the solid support (often referred to as cleavage and deprotection). Subsequent filtration and washing (to remove the solid support) result in a solution of crude, 5′-DMT protected oligonucleotide, ready for purification

The crude oligonucleotide solution is purified by liquid chromatography, typically strong anion ion exchange (SAX). Impurities not closely related to the active pharmaceutical ingredient (API; eg, by virtue of significant difference in chain length—shortmers/longmers) are readily separated during the chromatographic purification step; however, full-length (and close to full length) oligonucleotide impurities will not be removed during this step. The eluate is progressed forward to the desalting/concentration step.

Depending on the precise nature of the process, the 5′-DMT group can be removed during the solid-supported synthesis before amine treatment, during chromatography, or as a standalone postchromatography operation.

In the desalting/concentration step the counter ion is exchanged (if needed) and the oligonucleotide solution is concentrated. This can be achieved by ultrafiltration/diafiltration, for example, through use of a tangential flow filtration apparatus equipped with membranes. During this step, residual organic solvents, salts, and low-molecular weight impurities are removed according to the pore diameter cutoff size of the membrane. Alternatively, it is possible to carry out a sequence of ethanol-based oligonucleotide precipitations and subsequent reconstitutions from water as a means to remove low-molecular weight impurities and concentrate the oligonucleotide.

After the desalting and concentration operations, the API can be provided directly as an aqueous concentrate [5] or isolated as a solid—typically by lyophilization.

The repetitive nature of this overall oligonucleotide synthesis process where individual cycles apply similar conditions to substrates and reagents of broadly similar reactivity delivers a predictable outcome and is routinely used as the method of choice for Good Manufacturing Practice (GMP) manufacture of therapeutic oligonucleotides. In this respect, the high degree of automation, absence of in-process testing, and robust performance are reminiscent of a well-understood, well-defined commercial manufacturing process, even for preclinical manufactures.

Although oligonucleotide therapeutics are explicitly excluded from the scope of ICHQ6A (and by reference to ICHQ11), there are concepts in the quality guidelines that are currently being generally applied to oligonucleotide manufacturing with the most relevant being ICH M7 (regarding mutagenic impurities) [6], ICH Q3C (residual solvents) [7] and ICH Q3D (elemental impurities) [8], and ICH Q7 (Good manufacturing practices) [9]. One notable diversion from the guidance relates to ICH Q3A (Impurities in New Drug Substances [10]). While the general concepts of reporting limits, identification limits, and qualification limits still apply, higher thresholds have been accepted for oligonucleotides than for small molecules, to date [11].

ICH Q11 provides a science-driven, risk-based framework addressing the propensity for SMs to influence the quality of the drug substance. This requires a thorough understanding of actual and potential impurities, as well as their fate in downstream processing gained from knowledge of the synthetic route coupled with risk assessments. Although ICH Q11 explicitly states that oligonucleotides are out of scope, the ethos for SM selection outlined in ICH Q11 remains applicable, but should be considered with an appreciation of oligonucleotide processing. Working in this way, a number of principles can be considered in the designation of oligonucleotides SMs:

- A defined and stable structure with characteristic chemical and physical properties
- A significant structural fragment toward the structure of the drug substance
- Oligonucleotides with related sequences or size typically possess similar physical properties; so purging of impurities with the same or similar number of nucleotides as the desired product (full length impurities) is challenging.
- Effective SM specifications supported by detailed understanding of the fate and control of impurities in phosphoramidites or other SMs are vital aspects of the overall oligonucleotide control strategy.
- Analysis of the risk of impurity carryover across the unit operations is of greater priority than number of chemical transformations to reduce the risk of contamination and support the control strategy throughout the product lifecycle.
- The operation of many steps without interspersed analysis during solid-supported synthesis does not support application of traditional stepwise impurity fate and purging approaches.
- Ideally, the SM can be sourced as a commodity with controlled quality

Phosphoramidite building blocks (henceforth described as amidites) are manufactured using standard chemical manufacturing technology and are well controlled. Several amidites are widely available from third-party commercial suppliers with controlled quality. To date, 2′-deoxyribose amidites (deoxyamidites) and 2′-(2-methoxyethoxy)ribose amidites (MOE amidites) have been accepted as appropriate SMs for oligonucleotides, demonstrating that amidites can be acceptable in accordance with the principles of ICH Q11 guidelines. In this situation, the application of GMPcontrols for oligonucleotide drug substance manufacturing processes starts from the amidite SMs with an appropriate control strategy to ensure quality of the finished oligonucleotide.

### Deoxyamidites

**FIG. 3.**
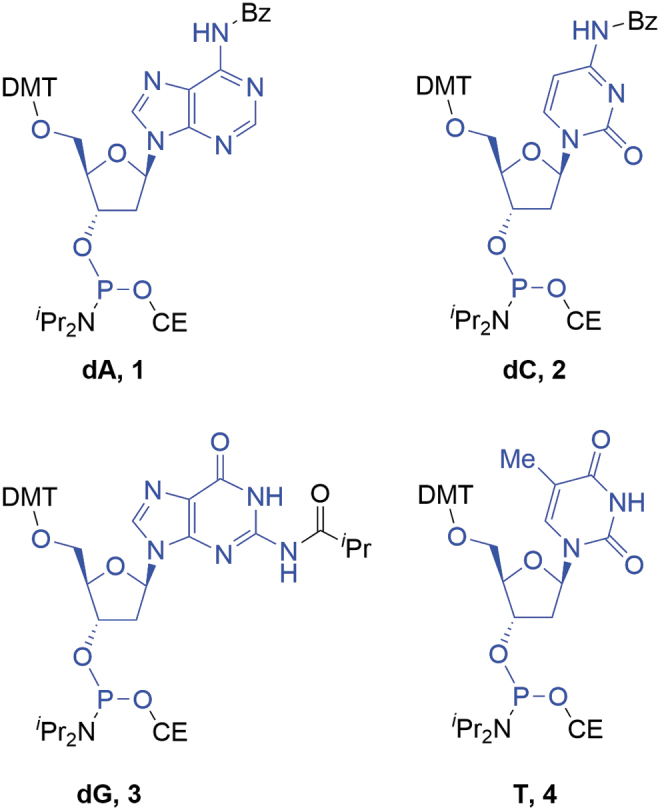
Deoxyamidites (atoms marked in *blue* are incorporated into the oligonucleotide).

The deoxyamidites, for example, **1–4** (Fig. 3), whose core structures are found naturally in DNA, are the most widely used building blocks in oligonucleotide APIs. As such, they provide a convenient introduction for how regulatory guidance can be applied to oligonucleotide SMs before introducing more complex examples.

Deoxyamidites are incorporated into the oligonucleotide as significant structural fragments and therefore fulfill the most basic requirement for SMs. The most prevalent examples are the 2-cyanoethyl-*N*,*N*-di*iso*propylaminophosphoramidites, which were first applied in oligonucleotide synthesis in 1984 [12]. They were rapidly adopted as the standard approach due to supporting highly efficient coupling following appropriate activation. Supply of these materials has increased to a point where they are commercially available in large quantities (up to hundreds of kg batch size) from multiple vendors worldwide with multiton annual capacity available in the market. In addition, there are many more vendors capable of supplying medium- to small-scale amounts of material.

As might be expected for such established materials, the structure and physiochemical properties of deoxyamidites have been rigorously characterized. A variety of analytical techniques, for example, ^1^H/^13^C/^31^P/2D-NMR (two-dimensional nuclear magnetic resonance spectroscopy), specific rotation, and high-performance liquid chromatography with ultraviolet and mass spectrometry detection (HPLC-UV-MS) have been applied in EPOC member companies and elsewhere, providing a confidence in the robustness of their quality. These studies have led to a detailed understanding of potential and actual impurities, as well as their fate and impact on the quality of the target oligonucleotide. Consequently, the material attributes of the deoxyamidites that may impact the API critical quality attributes (CQAs) [1] (eg, reactive critical, impurities) can be defined, and analytical methods with appropriate acceptance criteria can be validated, leading to specifications to purchase materials. This comprehensive understanding further supports acceptance of deoxyamidites as SMs, and a more detailed discussion is presented in the next section.

**FIG. 4.**
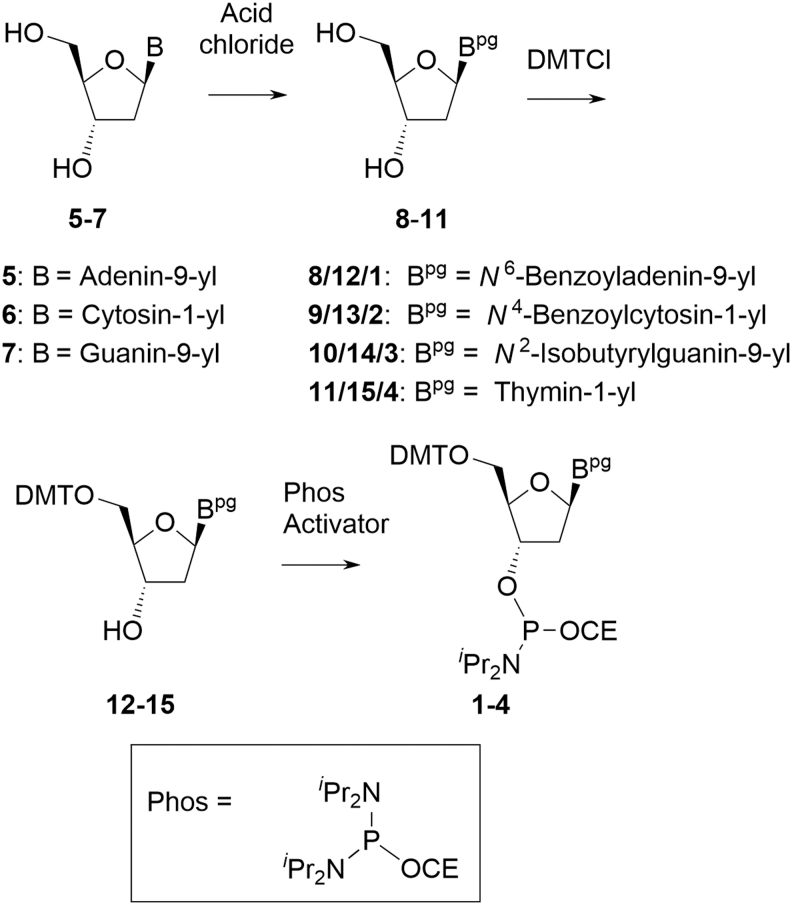
General method used to manufacture deoxyamidites.

Deoxyamidites are free-flowing, nonhygroscopic, amorphous solids. They are derived from the corresponding nucleosides that are obtained from non-animal sources through fermentation and readily available in ton quantities in stereochemically pure form. The amidites are a mixture of the two diastereoisomers at the P atom and typically seen as double peaks both in ^31^P NMR and high-performance liquid chromatography (HPLC). Commercially available deoxyamidites are synthesized by the following general synthesis scheme (Fig. 4).

The 2′-deoxyribonucleosides **5–7** are acylated on the exocyclic primary amines of the nucleobases, that is, adenine, cytosine, and guanine, with desired protecting groups (step 2). Since thymine does not have an exocyclic amine, this step is not performed. Protection of the 5′-hydroxyl as the DMT ether affords the fully protected nucleosides (**12–15**, often referred to as PNS). The final step is the phosphitylation of PNS with 2-cyanoethyl-*N*,*N*,*N′*,*N′*-tetra*iso*propylphosphordiamidite (often referred to as Phos reagent or P-reagent) in the presence of an activator. Preferred activators are small, weak, nonhygroscopic organic acids.^†^ Although no systematic study has been conducted, anecdotal evidence derived from more than 20 years of experience within the EPOC partners related to deoxyamidites synthesized using two common activators (1*H*-tetrazole, DCI) indicates that activator choice does not appear to influence the amounts of reactive impurities that result in oligonucleotide drug substance impurities. For similar reasons, solvents used in the reaction are not considered to impact SM CQAs.

In current commercial scale processes, although no new carbon stereocenter is created from the nucleoside, the synthesis of the amidite results in an *R*/*S* stereochemical mixture at phosphorus. Crucially, the absolute configuration of the phosphorus atom of deoxyamidites does not impact the distribution of oligonucleotide diastereoisomers. This is determined by other variables during the coupling reaction in the oligonucleotide chain extension cycle; therefore, control of phosphorus stereochemistry in deoxyamidites is unimportant [13,14].

**FIG. 5.**
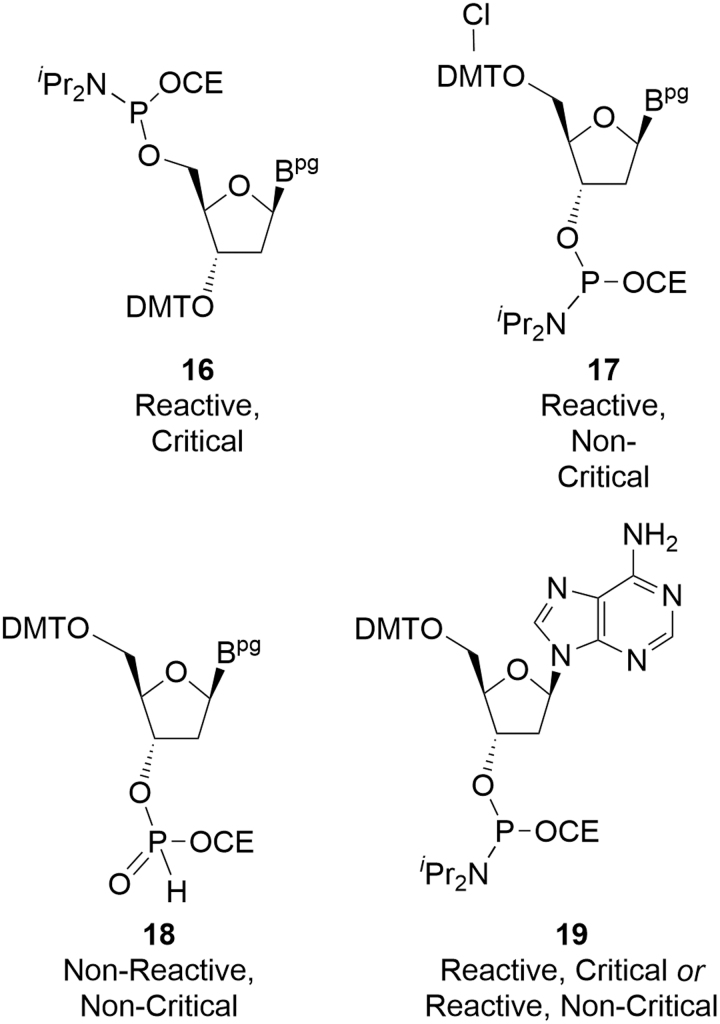
Examples of amidite impurity types.

Impurities in deoxyamidites can be assigned to two broad groups based on an assessment of their reactivity during oligonucleotide coupling and the ability to purge any resulting impurity during the manufacturing process. The most important group results in impurities in the crude drug substance that are not subsequently purged. These are known as reactive, critical (or critical) impurities and generally contain both phosphoramidite functionality and an acid-labile protecting group, such that they can propagate chain elongation (Fig. 5). It is the individual and total amounts of these critical impurities that constitute the CQAs of deoxyamidites [15].

The second group comprises deoxyamidite impurities that have no impact on the final drug substance purity and, unsurprisingly, these are commonly known as noncritical impurities. These might be such species as related nucleosides and nucleotides or residual solvents that do not react with the oligonucleotide chain during coupling. Such inert components are known as nonreactive, noncritical impurities. There is a second subset of noncritical impurities that do react with the evolving oligonucleotide chain during coupling, but do not affect product quality. This might be due to the resulting impurity in the oligonucleotide being readily purged, for example, during chromatography, or because the impurity motif is lost during processing and results in the target product. These are the reactive, noncritical impurities. Examples of each class are presented (Table 1).

**Table 1.** Origin and Control of Indicative Critical and Noncritical Impurities in Deoxyamidites

Impurity type	Source	Method(s) of control
**16**	Reaction of DMT-Cl with 3′-OH rather than 5′-OH during PNS formation	PNS purification; deoxyamidite purification; deoxyamidite specification
Reactive, critical		
**17**	Impurity in DMT-Cl reagent with one chlorine on one aryl ring	Vendor DMT-Cl specification; deoxyamidite specification
Reactive, noncritical		
**18**	Amidite hydrolysis during phosphitylation	Control of water during PNS phosphitylation; deoxyamidite purification; deoxyamidite specification
Nonreactive, noncritical		
**19**	Bz protecting group was either never installed or was installed, but later cleaved	PNS purification; deoxyamidite purification; deoxyamidite specification
Reactive, critical or reactive, noncritical		

DMT, 4,4′-dimethoxytrityl; PNS, protected nucleoside.

The reactive, critical impurity **16** will incorporate into the oligonucleotide during the coupling reaction through a 5′-5′ internucleotide linkage. Detritylation during the subsequent deprotection step will actually release a terminal 3′-hydroxyl at the expected 5′-terminus leading to a subsequent 3′-3′ internucleotide linkage before reverting to normal progress in later cycles. This introduces a reversed nucleotide to a sequence. Due to its similarity to the parent, oligonucleotides containing **16** are not purged during downstream processing, thereby rendering it critical; **16** is controlled by purification at the PNS and amidite stages and by specification.

It is important to note that the deoxyamidite examples provided in Table 1 were used to illustrate the definitions of the classes of impurities, but in actual practice, there are almost no traces of critical impurities in deoxyamidites. Specifically, the levels of **16**, when present at all, are very low (∼0.04%) due to the high degree of selectivity between the 3′- and 5′-hydroxyls in the tritylation reaction.

The presence of an extra chlorine atom in reactive, noncritical impurity **17** does not materially affect the rate of subsequent detritylation, which removes this impurity motif from the chain. Oligonucleotide chains that have incorporated **17** continue to elongate into full-length parent oligonucleotide. Therefore, while impurity **17** is reactive during the synthesis, it is noncritical with respect to oligonucleotide CQAs.

The nonreactive, noncritical impurity **18** (H-phosphonate) is unreactive under normal amidite coupling conditions and therefore passes through the synthesis column and away from the oligonucleotide during amidite delivery and subsequent washing steps. While noncritical, some control over **18** is advantageous for yield consistency.

Criticality for other reactive impurities will not always be so clearly defined. Reactive impurity **19** might be present in **1** and will couple as normal, but will introduce branching impurities throughout the rest of the synthesis. During subsequent coupling cycles, one or both growing branches may fail to extend and be capped, generating a complex mixture of branched species of various lengths with as many as two DMT groups present on the 5′-termini. Depending on which coupling cycle was compromised, the resulting mixture of branched oligonucleotide impurities may be easier or harder for the chosen downstream purification method to purge. Incorporation in the first coupling cycle would result in a much larger (and generally easier to separate) oligonucleotide impurity than for the final cycle where it may just lead to parent oligonucleotide. In such cases, criticality will need to be assessed for the process chosen and the appropriate limit set for **19**.

Deoxyamidites typically contain trace quantities of water and solvents (eg, acetonitrile, ethyl acetate, and toluene). These do not react with the growing support-bound oligonucleotide and are washed away or purged, as such they do not affect drug substance purity and are therefore considered noncritical. A steady evolution of deoxyamidite manufacturing means that there are almost no traces of critical impurities observed (Table 2).

**Table 2.** Deoxyamidite Impurity Lot History Summary

Vendor	No. of lots	Lots with critical impurities	Assay range (% a/a)	^31^P purity range (% a/a)	Water (% w/w)	Residual solvents (% w/w)
1	31	8	94.0–99.6	96.8–100.0	0.11–0.35	0.23–2.90
2	22	5	94.4–99.7	98.8–100	0.07–0.67	0.84–2.42
3	10	1	97.0–99.5	99.3–99.9	0.10–0.28	0.22–2.36
4	9	1	94.9–100.5	97.9–100	0.11–0.23	ND–1.1
Total	72	15				

ND, not detected.

Of 72 lots of materials manufactured by 4 vendors on multi-kg scales, 13 contained a single critical impurity in the range 0.04%–0.11% with a further 2 batches containing only 19 in the range 0.2%–0.3%. Only two batches contained >1 critical impurity, but still totaling <0.2%. Similar summary statistics on assay, purity by ^31^P NMR, water content, and residual solvents (Table 3) show deoxyamidites to be of consistently high quality from multiple vendors.

**Table 3.** Additional Summary Statistics for 72 Lots of Deoxyamidites

Characterization	Mean	Median	SD
Assay (% w/w)	97.5	98.1	1.62
Purity by ^31^P NMR (% a/a)	99.2	99.4	0.83
Water (% w/w)	0.19	0.15	0.108
Residual solvents (% w/w)	1.07	0.95	0.568

NMR, nuclear magnetic resonance spectroscopy.

Specifications for deoxyamidites focus on control of critical impurities and overall purity (Table 4). The need to control critical impurities will be evident from the foregoing discussion, but control of overall purity can also be important. For example, reversed-phase purification ruggedly purges failure sequences, whereas with some oligonucleotide sequences, strong anion exchange chromatography is sensitive to quality of input materials. If the chosen process for oligonucleotide manufacture cannot completely purge coupling failures, then coupling efficiency becomes a critical process parameter and, by extension, the overall purity of the deoxyamidites becomes critical. Even if the oligonucleotide process can purge all failed sequences, it is still advantageous to control the overall deoxyamidite purity to aid process robustness (and to avoid paying for expensive noncritical impurities). When considering critical impurities, it is also necessary to consider the multiplicity of deoxyamidite incorporation due to the impurity family approach typically applied to oligonucleotide impurities [11]. The presence of a single, critical reactive amidite impurity at 0.05% w/w in each amidite during the synthesis of a 20-mer oligonucleotide could lead to a maximum of 1.0% (20 × 0.05) of the corresponding oligonucleotide impurity. This is due to amplification depending upon how often the individual amidite is used in a specific sequence since the motif will be incorporated at low level during each coupling cycle. This may not be a problem if amidite impurities are qualified appropriately, but could lead to surprises if not anticipated and controlled in the deoxyamidite specification.

**Table 4.** Example Specification For Deoxyamidite **1**

Test	Method	Acceptance criterion
Appearance	Visual inspection	White to yellow powder
Identification	LC-UV-MS	MoIM of the sample and the reference standard agree to within an amu limit. Retention times of both main peaks of the sample and reference standard agree to within a limit
Assay	LC^a,b^	NLT 90.0%
Impurity profile	LC^a,b^	Critical impurity
		NMT 0.20%
		Any unspecified critical impurity^c^
		NMT 0.15%
		Total critical impurities
		NMT 0.50%
Purity	^31^P NMR	≥95.0% a/a
Water content	KF	NMT 1.0% w/w
Residual solvents	GC	NMT 4.0% w/w

^a^ LC methods may be reported as either area percent or weight percent at a specific wavelength that can vary depending upon the method.

^b^ Both UV and MS detection methods have been applied.

^c^ Any impurity that contains both an amidite moiety and DMT protecting group may be critical and should be investigated; all other impurities are noncritical.

GC, gas chromatography; KF, Karl Fischer; LC-UV-MS, liquid chromatography with ultraviolet and mass spectrometry detection; MoIM, monoisotopic mass; NLT, not less than; NMT, not more than.

### More complex amidites

**FIG. 6.**
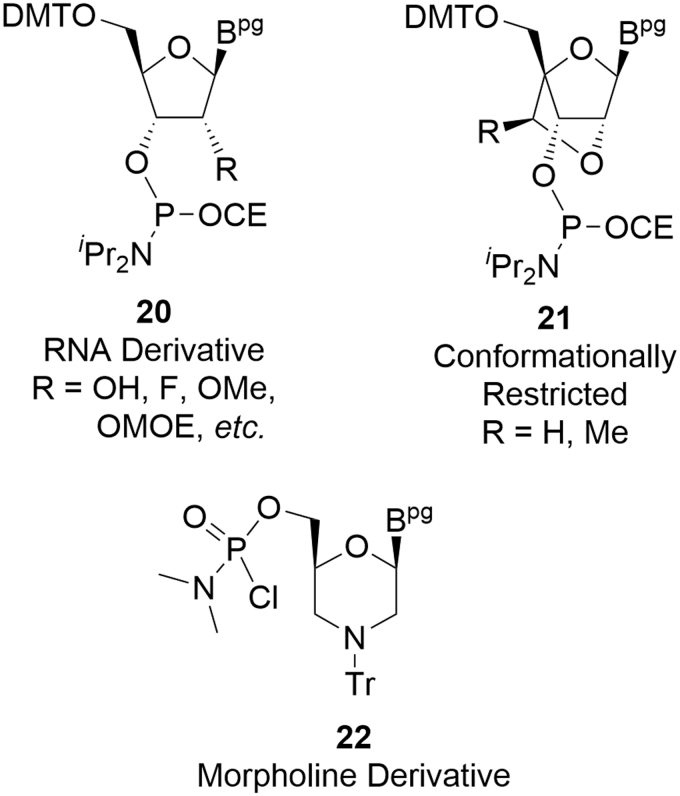
Examples of more complex amidites.

As the oligonucleotide space has matured, a broader range of amidites has been accommodated within sequences (Fig. 6). The more popular of these have been those derived from RNA nucleotides **20** such as 2′-F [16] and 2′-OMe [17], more elaborate versions such as the LNAs **21** [18], and other ring systems such as the morpholinos **22** that lead to the phosphomorpholino oligonucleotides (PMOs) [19], as well as many others. It should come as no surprise that the same approaches and impurity classifications identified for the deoxyamidites can be applied to these more complex amidites.

**FIG. 7.**
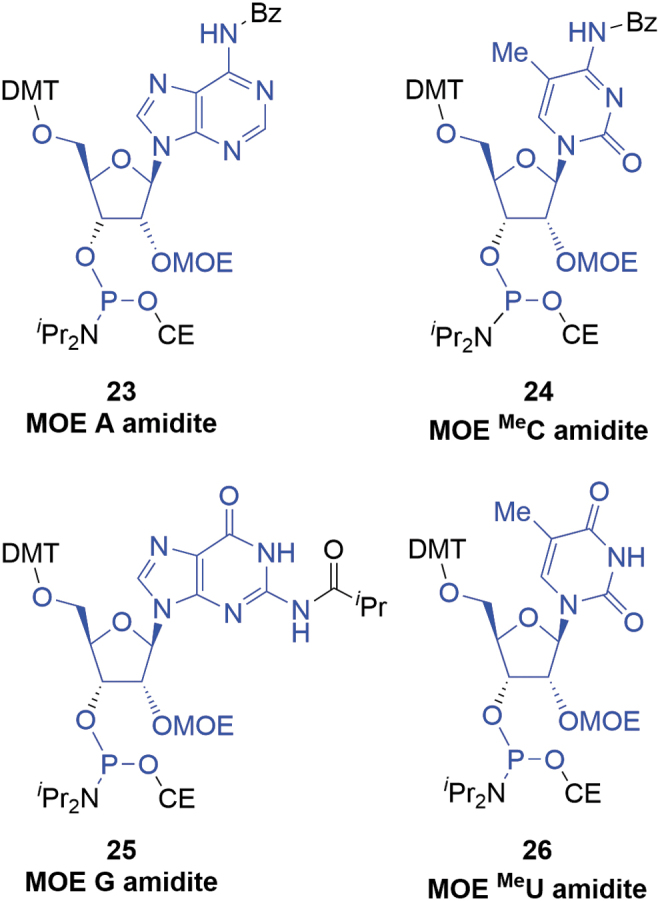
MOE amidites (atoms marked in *blue* are incorporated into the oligonucleotide). MOE, 2′-(methoxyethoxy)ribose.

One of the most popular ribose-derived monomers used in oligonucleotide APIs are the 2′-*O*-(2-methoxyethyl)ribonucleoside amidites (MOE amidites, **23–26** R = OMOE) (Fig. 7). MOE amidites are SMs for a number of marketed products (Kynamro, Tegsedi, Spinraza, and Waylivra) and will be used to extend the deoxyamidite argument into a more complex case.

**FIG. 8.**
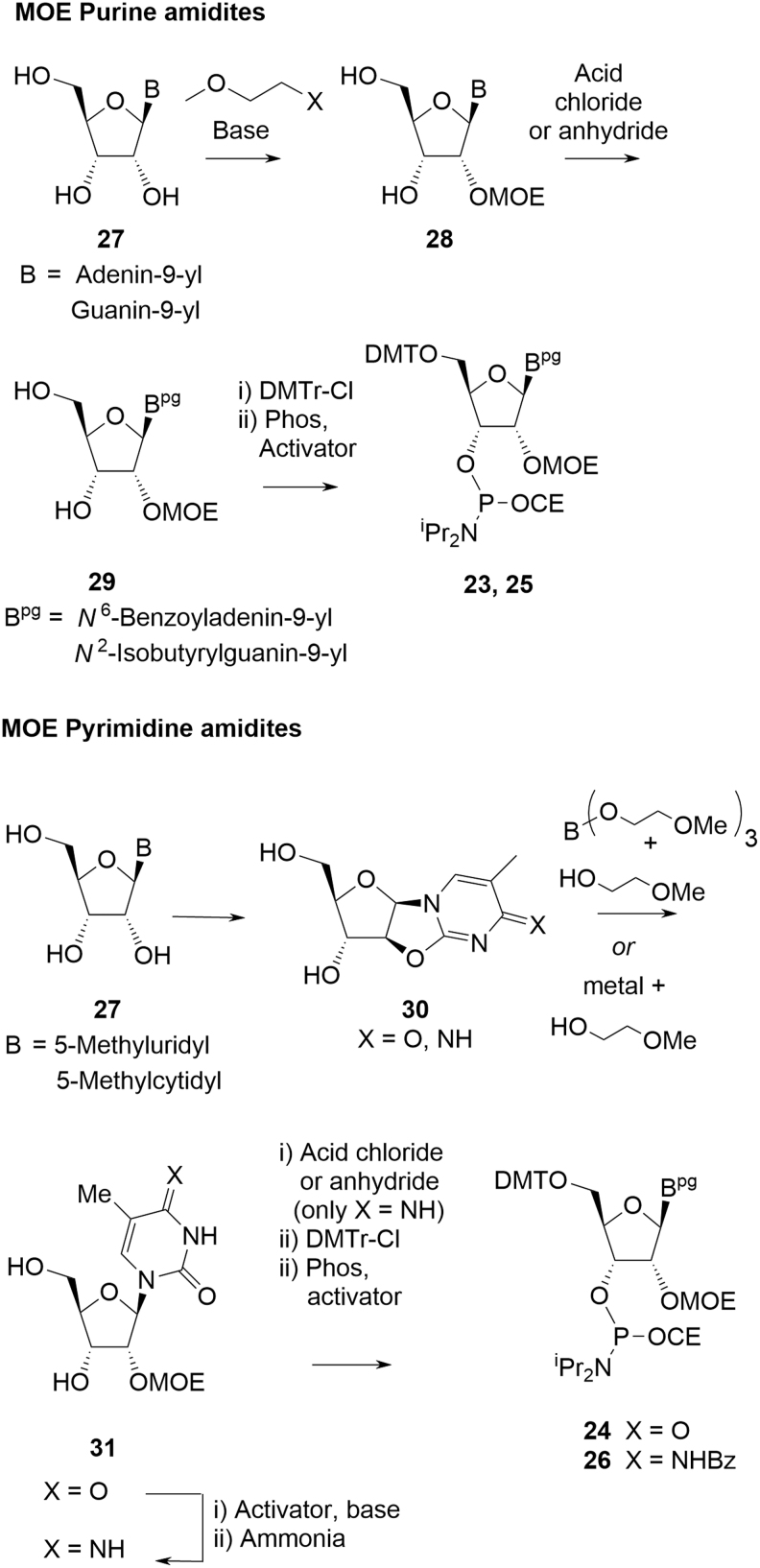
General approaches to MOE amidites.

These materials are coupled in the oligonucleotide synthesis in the same manner as deoxyamidites. They are also stable solids and their raw materials (non-animal sourced nucleosides **27**) are available in ton quantities. The synthesis of commercially available MOE amidites follows a general approach dependent on whether they bear purine or pyrimidine bases (Fig. 8).

Introduction of the MOE group onto the 2′-hydroxyl of nucleoside **27** is achieved in one of two ways. If **24** possesses a purine nucleobase (ie, adenine and guanine), direct alkylation of the nucleoside at the 2′-hydroxyl is achieved with an activated form of 2-methoxyethanol [20]. Thus, analogous to the deoxyamidites, all the ribose stereocenters of, for example, **23** and **25,** are derived directly from the corresponding sugar and stereochemical integrity is maintained during conversion to the amidites. Stereoisomers of **23** or **25** should, therefore, not be treated as CQAs.

When the nucleobase in **27** is a pyrimidine (eg, 5-Me cytosine and 5-Me uracil), it is first converted to a bicyclic oxazolidine **30,** inverting the stereochemistry at the 2′-position following an S_N_2 mechanism [21]; **30** can only be formed as a single stereochemical isomer following neighboring group displacement of the activated 2′-OH by the nucleobase. Ring opening of **30** with 2-methoxyethanol or a nucleophilic variant also results in inversion of C2′, that is, the overall effect can only be for double inversion at C2′, thus retaining the natural configuration.

For both ring types, nucleosides **28** and **31** are converted into the corresponding MOE amidites in the same way as for deoxyamidites. The exocyclic primary amines of the nucleobases are acylated (adenine, methylcytosine, and guanine), the 5′-hydroxyl is protected as the DMT ether, and finally, the 3′-hydroxyl is phosphitylated with Phos reagent in the presence of an activator. For pyrimidines, nucleoside **31** (X = O) can also be converted into **31** (X = NH) if required.

**FIG. 9.**
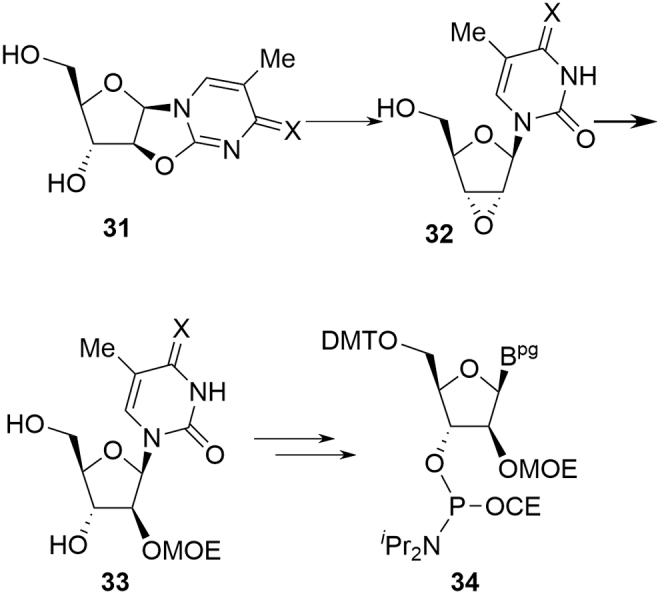
Theoretical C-2′ inversion of MOE amidite precursors.

In the case of pyrimidine MOE amidites, there is a theoretical risk that **30** could be subject to an alternative reaction involving neighboring group attack from the 3′-hydroxyl leading to epoxide **32** (Fig. 9). In this situation, epoxide opening of **32** by methoxyethanol would give **33** as an impurity in **31,** which, on completion of the synthesis, would be expected to provide **34** as a critical, diastereomeric MOE amidite impurity. As with all such investigations, it is always important to ensure that the potential for impurity formation through reasonable reaction pathways has been considered. In this case, the isomer pathway leading to **34** has never been observed, despite extensive investigations (McPherson A, 2020, unpublished data), although it might be viewed as prudent to demonstrate specificity for **34** in the release methods, for example, **24** and **26**. As a consequence of this observation and, since the integrity of all other stereocenters remains intact during conversion of **27** to **24** or **26**, the stereochemical integrity of pyrimidine MOEs is not treated as a CQA.

**FIG. 10.**
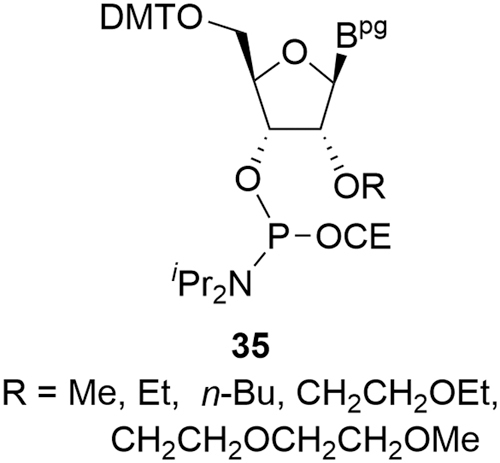
2′-*O* impurities in MOE amidites.

In the same way as for deoxyamidites, MOE amidite impurities can be treated as noncritical or critical and the same conditions apply. Species that do not react (eg, solvents, phosphonates, phosphonoamidates, and phosphoramidates), either do not contain an activatable amidite group or are analogous to the reactive, noncritical deoxyamidite impurities described earlier and are readily removed during purification. Introduction of the alkyl side chain at the 2′-*O* position does introduce some 2′-*O* impurities **35** that can be incorporated into the oligonucleotide during synthesis and should be considered critical impurities (Fig. 10). These alkylation impurities are monitored in the MOE amidites by HPLC-UV-MS and are readily controlled in the SM specifications.

**FIG. 11.**
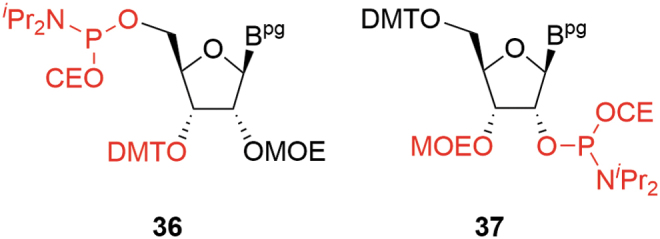
Regioisomeric MOE amidite impurities.

As with deoxyamidites, MOE amidites carry the potential for regioisomeric impurities **36** (sometimes called inverted amidites) if tritylation occurs on the 3′-hydroxyl and phosphitylation occurs on the 5′-hydroxyl (Fig. 11). Furthermore, an alternative 3′-alkylation of purine MOE nucleosides could occur followed by 2′-phosphitylation to form a different set of regioisomer impurities **37**. Any oligonucleotide impurity derived from **36** and **37** would be not be removed during downstream processing due to the similarity with the parent; **36** and **37** are therefore controlled by purification in the MOE amidite SM synthesis and by SM specifications.

As might be expected from the additional manipulations in their synthesis, MOE amidites typically have more critical impurities than their deoxyamidite analogs. Of 104 lots of materials manufactured on multi-kg scales by 4 vendors, critical impurities ranged from below the limit of detection (<0.04% a/a) to a high of 0.8% a/a (Table 5). Similar summaries on assay, purity by ^31^P NMR, water content, and residual solvents show that MOE amidites are manufactured to a consistently high quality from multiple vendors.

**Table 5.** MOE Amidite Impurity Range Summary

Amidite	No. of lots	Assay (% w/w)	Purity by ^31^P NMR (% a/a)	Total critical impurities (% a/a)	Water (% w/w)	Residual solvents (% w/w)
MOE ^Me^U	24	91–101	95–100	0.04–0.8	0.1–0.5	0.3–1.7
MOE ^Me^C	28	95–101	97–100	0.04–0.25	0.1–0.3	0.2–2
MOE A	25	97–101	97–100	0.04–0.4	0.1–0.7	0.3–1.7
MOE G	27	92–98	96–99	0.04–0.7	0.2–0.9	0.3–3.2

MOE amidites in Table 6 have the base protection schemes depicted in Fig. 5.

MOE, 2′-(methoxyethoxy)ribose.

**Table 6.** Example Impurity Specifications for MOE Amidites

Test	Method	Acceptance criterion	
Impurity profile	HPLC^a^	Critical Impurity	NMT 0.2–0.4% a/a
		Any unspecified critical impurity^b^	NMT 0.15% a/a
		Total critical impurities	NMT 0.5–0.8% a/a
Purity	^31^P NMR		NLT 95.0% a/a

^a^ Both UV and MS detection methods have been applied.

^b^ Any impurity containing both a amidite moiety and a DMT protecting group may be critical and should be investigated; all other impurities are noncritical.

HPLC, high-performance liquid chromatography.

Generally, MOE amidite specifications track water content and solvents similar to those for deoxyamidites, but mainly focus on control of critical impurities and overall purity. Typical values for impurity limits used in clinical and commercial products by EPOC partners are outlined (Table 6) (Note: limits should be defined and be fit for each new oligonucleotide sequence and tailored to the controls for the specific manufacturing process—one size does not fit all). The specifications for individual critical impurities and the totals are somewhat higher than for the corresponding deoxyamidites. This reflects that higher levels of detectable impurities have been observed and used successfully in clinical and commercial manufacturing by EPOC partners.

All the points raised for deoxyamidites and MOE amidites can be extended further for even more complex amidites. These are often proprietary in nature, which brings the additional complication that supply chains may not be so well established as the deoxyamidites and MOE amidites. In addition, multiple synthetic routes might be employed to deliver materials.

The LNA derivatives are typical examples of this additional complexity and, in the case of the more challenging constrained ethyl (cEt) amidites (cEts, **21** R = Me) (Fig. 6), a number of chemical routes to these materials have been published [22–24]. A common characteristic is that all follow lengthy synthetic sequences, although the final stages are similar.

**FIG. 12.**
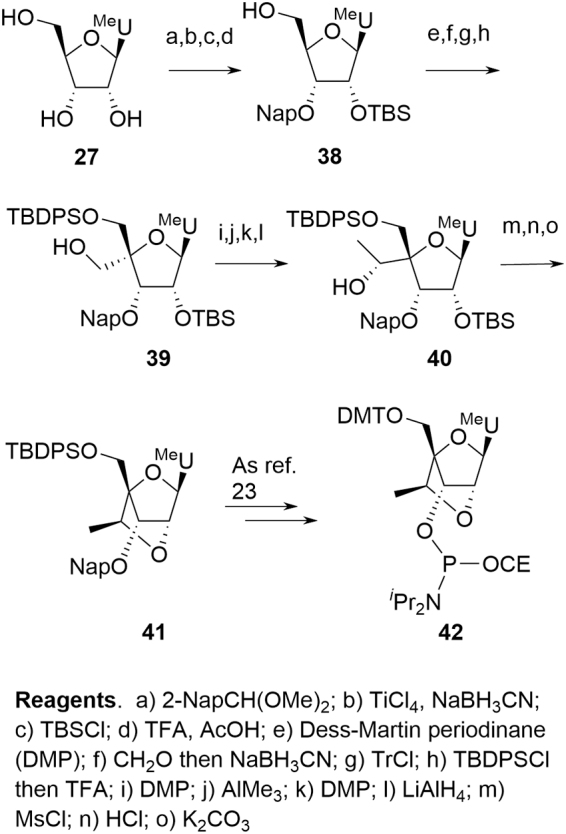
Salinas cEt route.

Salinas *et al*.'s approach [23] (Fig. 12) is a linear synthesis and starts from the appropriate RNA nucleoside **26** in a similar manner to the MOE amidites. Although only demonstrated for cEt ^Me^U **42**, extension to other amidites should be possible.

**FIG. 13.**
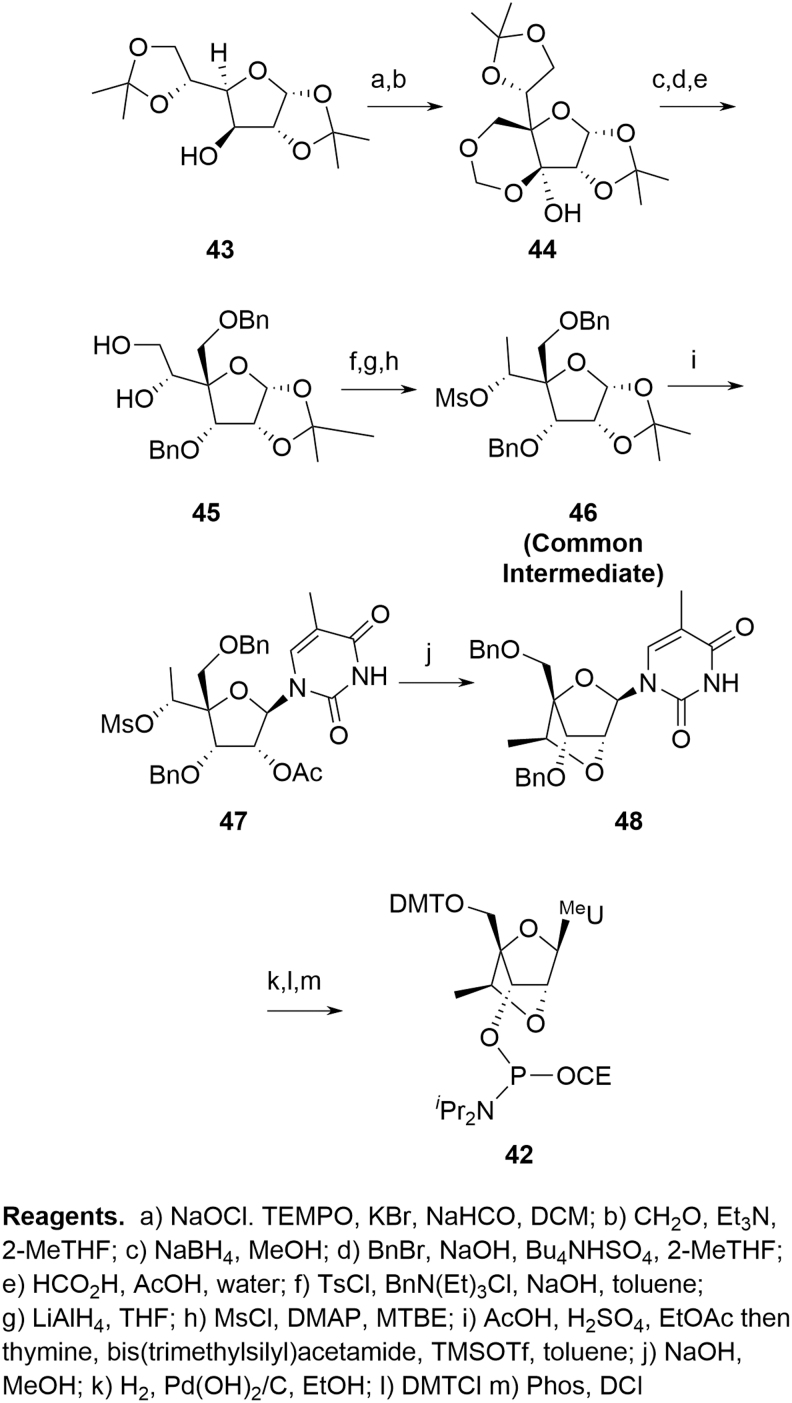
Blade cEt route.

The Seth *et al*. [22] and Blade *et al*. [24] syntheses offer a more complete approach to delivering a range of cEt amidites. In both cases, a linear sequence provides a common intermediate such as **46** (Fig. 13), which supports a divergent approach to the required amidites; **46** contains the key skeletal elements present in the final amidite, although not in the final, structural presentation. Given acceptable molecular properties, **46** also represents a convenient storage point if flexibility is required, for example, to support a broad development portfolio. The Salinas synthesis is identical to that of Seth's from **39** onward. A similar divergent approach is applied toward the LNAs (**21**, R = H) [25,26].

The nature of the route (divergent vs. linear) is important since critical reactive impurities would generally be expected to possess comparable kinetics to the required phosphoramidites during coupling. The linear approach reflects a more traditional situation for SMs where provision of each cEt amidite can be viewed as an independent activity with impurity identification and purging only relevant to that specific SM.

In the case of the divergent approaches, the situation is somewhat more complex. Any inherent impurity not purged before isolation of **46** could, in principle, lead to analogous impurities in all cEt amidites generated from that batch, and in turn result in higher levels of the corresponding oligonucleotide impurity. Impurities generated downstream from **46** will be discrete to the individual cEt amidites, although the chemistry across the divergent stages is quite similar and therefore one might see common issues to various extents.

**FIG. 14.**
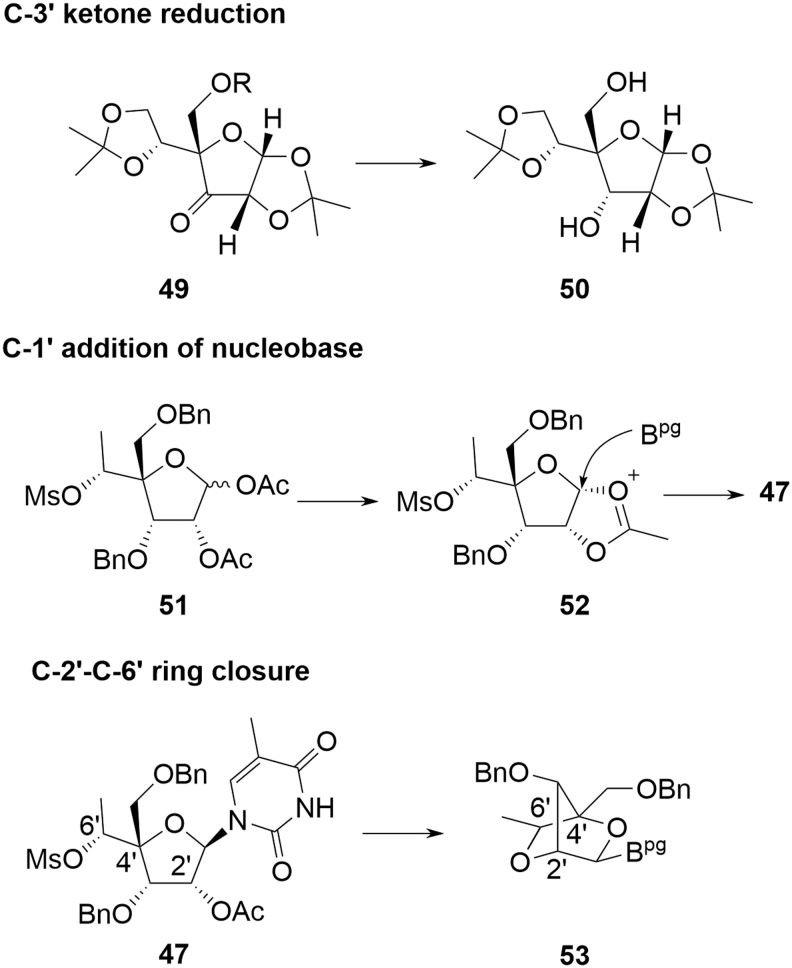
Stereochemical control in cEt amidites.

In practice, the linear approach is not pursued at scale and cEt amidites are produced by one or other of the divergent syntheses. As with deoxyamidites and MOE amidites, the precursor di(acetone)glucose is chiral, naturally derived, and well characterized with unambiguous stereochemistry. A key difference in the case of the cEt amidites is that reaction occurs at four of the five original stereocenters with overall inversion at each, potentially resulting in a more complex chemistry to follow for the SM. Confidence in stereochemical integrity can be provided by approaches such as X-ray structural elucidation or NMR correlation studies following key transformations. Some general mechanistic observations can also be applied, however, which further mitigate the potential impact of this apparent complexity:

- The stereochemistry at C-3′ is inverted from that originally in di(acetone)glucose as a result of oxidation and later reduction. This is a common reaction sequence on protected glucose to invert C-3′ and, hence, obtain less common sugars [27]. Steric crowding ensures that delivery of hydride during reduction of **49** (Fig. 14) occurs from the convex face of the [3.3.0] ring system, resulting in the desired 3′-(*S*) configuration in **50**.
- The base is added through a Vorbrüggen reaction [28,29], whereby an equilibrating mixture of activated C-1′ acetate isomers **51** is internally displaced by participation of the C-2′ acetate, giving acetoxy-bridged intermediate **52** (Fig. 14). This is a well-documented process and is followed by opening of **52** at the activated C-1′ by the nucleobase leading to **47**. This again occurs from the convex face of a [3.3.0] bicyclic system resulting in the desired 1′-(*R*) configuration.
- Hydrolysis of the C-2′ acetate in **47** induces nucleophilic attack of the oxyanion on to the C-6′ mesylate to provide the cEt bicyclo [2.2.1] framework **53** (Fig. 14). This mandates the relationship between C-2′ and C-4′ since both the 2′-OH and the mesylate-bearing C-4′ side chain must be on the same face to successfully react. Since C-2′ retains the natural configuration found in glucose, this provides additional confidence for the stereochemistry at both C-2′ and C-4′. Also, in this step, S_*N*_2 displacement of the C-6′ mesylate sets the stereochemistry at C-6′ by inversion of the natural glucose stereochemistry.

Over and above the previously described concerns for deoxyamidites and MOE amidites, the major novel challenges for cEt amidites arise from the following:

- Control of stereochemistry for the pendant 6′-Me on the 2′-4′ bridging group
- 6′-Me-deletion impurity (*M-14*, **55**).

**FIG. 15.**
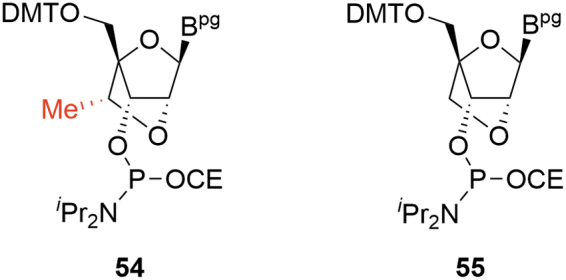
Critical cEt amidite impurities.

For all approaches, there is potential for low levels of the 6′-(*R*) diastereomer **54** to be formed (Fig. 15). Dependent on the synthesis employed, this is either a consequence of incomplete stereochemical inversion during an oxidation/reduction sequence (**39**→**40)** or through activation of the secondary alcohol rather than the primary during an epoxide formation (**45**→**46**); **54** can be readily identified at the point of formation, but reacts in subsequent steps in a similar manner to the parent 6′-(*S*) isomer and the resulting impurities can be tracked through the synthesis.

Impurity **55** is a feature of both the Seth and Salinas approaches arising from incomplete reaction during methyl addition to an aldehyde (**39**→**40)**. Such an impurity is to be expected and the requirement for a demonstrable understanding of its fate should be anticipated. The Blade approach avoids this methylation step completely and a paper assessment had ruled out this impurity motif. Surprisingly, **55** was observed as a byproduct, proposed to result from an unanticipated bond cleavage mechanism. This observation further emphasizes the need for vigilance during SM synthesis as with the previous situation relating to the anticipated, but unobserved 2′-diastereomer for MOE pyrimidine amidites **34**. The importance of a detailed understanding of generation and fate of impurities rather than taking an assumed position cannot be overemphasized. Once such an impurity is observed and identified, actions can be taken to proactively purge if deemed appropriate to reach desired quality levels (eg, through reactive chemistry or recrystallization of an intermediate). The impact of purging on these impurities is presented (Table 7).

**Table 7.** Purging of Impurities **54** And **55** During Constrained Ethyl Manufacture

Stage	***54*** (% a/a)	***55*** (% a/a)
Initial formation	0.24–0.30	0.7–1.0
**26**	0.05–0.12	0.5
cEt amidite	ND	0.1–0.2

cEt, constrained ethyl.

### Alternative synthetic approaches to oligonucleotides

**FIG. 16.**
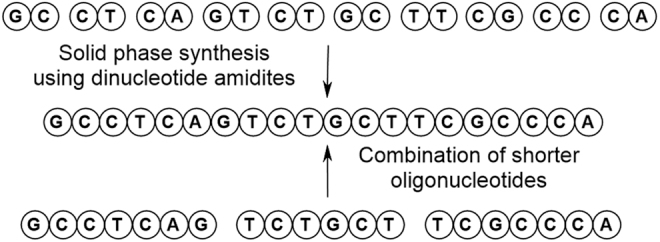
The use of blockmers in oligonucleotide synthesis.

In comparison with small molecule synthesis where convergent approaches are viewed as desirable, oligonucleotide synthesis has largely remained as a linear (and lengthy) exercise. In an effort to introduce convergency, alternative approaches have been considered such as the use of dinucleotide amidites [12,30–33] or the combination of shorter oligonucleotides as demonstrated using the templated ligation approach exemplified by Crameri *et al*. (Fig. 16) [34].

**FIG. 17.**
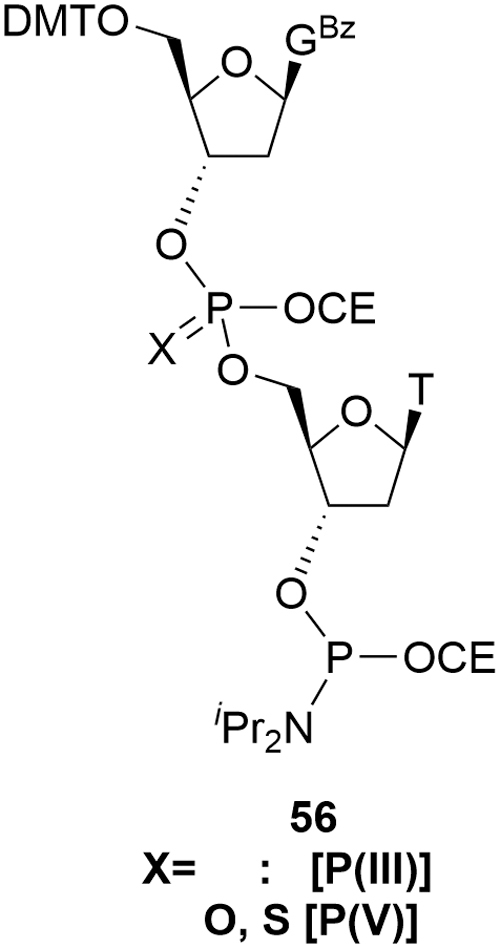
Representative blockmer deoxy amidite.

Such approaches are usually described as blockmers as exemplified by the deoxy GT blockmer amidite **56** (Fig. 17). Similar criteria to that described previously should be applied when designating SMs for these alternative approaches. A number of obvious additional challenges can be identified due to the existence of the internucleotide phosphorus linker. The first is associated with the coupling cycle required to deliver **56**. This results in a series of impurities more typically associated with finished oligonucleotides, for example, coupling failures, overcoupling, P = O (for X = S). Thus the set of reactive, critical impurities may be more significant than for the corresponding monomer deoxyamidites.

The presence of the second phosphorous functional group compounds this issue. The amidite group has already been identified previously as bringing a mixture of diastereomers. Aside from the special case of a stereo-specific phosphorothioate, the blockmer introduces a second, variable, chiral element leading to all discrete species being present as 4 diastereomers. In the case of a phosphate linkage, this complexity does not extend through to the API where the phosphate is achiral.

This additional stereochemistry has a dual impact since signal/noise is reduced and the number of potentially observable components increased, both by a factor of approximately two over standard monomer amidites. The consequence is that blockmers present greater technical challenges in identifying/quantifying impurities, thus increasing complexity during the development of an appropriate control strategy for these molecules. A blockmer approach will also require a larger number of potential SMs across a portfolio of projects rather than the limited number of monomer amidites generally employed to manufacture oligonucleotides.

No marketed oligonucleotides currently apply such convergent approaches, but as the pressure to supply ever larger quantities of oligonucleotides to meet growing patient demands continues to build, scalable alternatives to the current solid-supported manufacturing process may entertain these types of routes. The Q&A for ICH Q11 advises that convergent syntheses are acceptable and in answer to question 3, “ICH Q11 general principles apply to the selection of starting materials for linear or convergent syntheses [1]. The ICH Q11 general principles should be applied independently to each branch of a convergent synthesis, unless the point of convergence of the branches occurs upstream of an appropriate starting material.” In addition, although a blockmer contains multiple nucleotide subunits, they may be considered SMs as highlighted in answer to question 2, “ICH Q7 states that an ‘API starting material’ is a raw material, intermediate, or an API that is used in the production of an API. When a chemical, including one that is also an API, is proposed to be a SM, all ICH Q11 general principles still need to be considered.” More optimistically, it should be noted in the conceptually similar field of peptides that, at least one EPOC member company has reported successful justification of 2-mer peptides as SMs for the construction of larger peptides. In that case, several agencies accepted a specification for dipeptides that comprised comprehensive identity (composition and sequence), purity and impurity profile, chiral purity, water (Karl Fischer [KF]) or limit of detection (LoD), and residual solvents (A. Charaf, 2019, personal communication).

### Conjugating agents

**FIG. 18.**
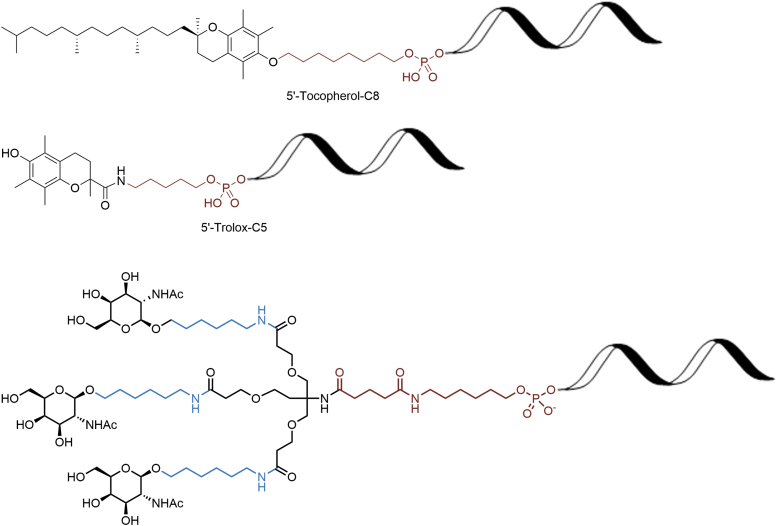
Selected small molecule conjugates.

**FIG. 19.**
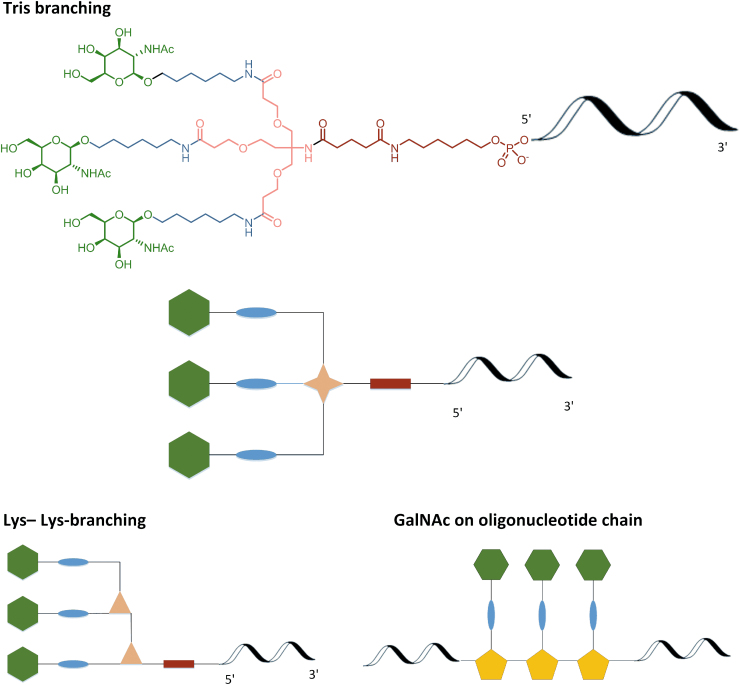
Selection of GalNAc attachment motifs. GalNAc, *N*-acetylgalactosamine.

Due to the cost of oligonucleotides, efforts to reduce patient dosage have become more important. Besides modified nucleotide chemistries, the use of conjugates has become more common to improve pharmacokinetics and distribution and facilitate cellular uptake mainly for antisense oligonucleotide (ASO) and small interfering RNA (siRNAs). The most common modifications are conjugate agents that are small molecules attached to the oligonucleotide (Fig. 18) such as cholesterol [35], tocopherol [36], anisamide [37], folic acid [38], peptides [39], anandamide [40], *N*-acetyl-d-galactosamine (GalNAc) [41], and poly(ethyleneglycol) ethers (PEGs) [42]. These are generally attached to the oligonucleotide through a cleavable tether often referred to as a linker (highlighted in red, Figs. 18 and 19). There is further activity, whereby large molecules such as antibodies [43] and aptamers [44] are applied, although this is out of scope for this discussion.

Most of these modifications can occur at either the 3′ or 5′ end of the oligonucleotide, although other modifications are also possible such as an internucleotide phosphonate [45].

*N*-acetyl-d-galactosamine (GalNAc) conjugation has become increasingly popular for the targeted delivery of chemically modified oligonucleotides to hepatocytes through binding to ASGR (asialoglycoprotein receptor) [46,47]. Although the chemical modification can take several forms, they retain a common feature, in that, several GalNAc moieties (typically 3—the triantennary structure) are connected to an oligonucleotide through a linker for optimal binding. Beyond this, a variety of differences in the nature of the spacer (eg, alkyl, ethylene glycol) and the point of attachment (eg, tris and lysine-lysine) to the oligonucleotide have been applied. These are schematically summarized (Fig. 19) for the most widely used approaches [48,49].

**FIG. 20.**
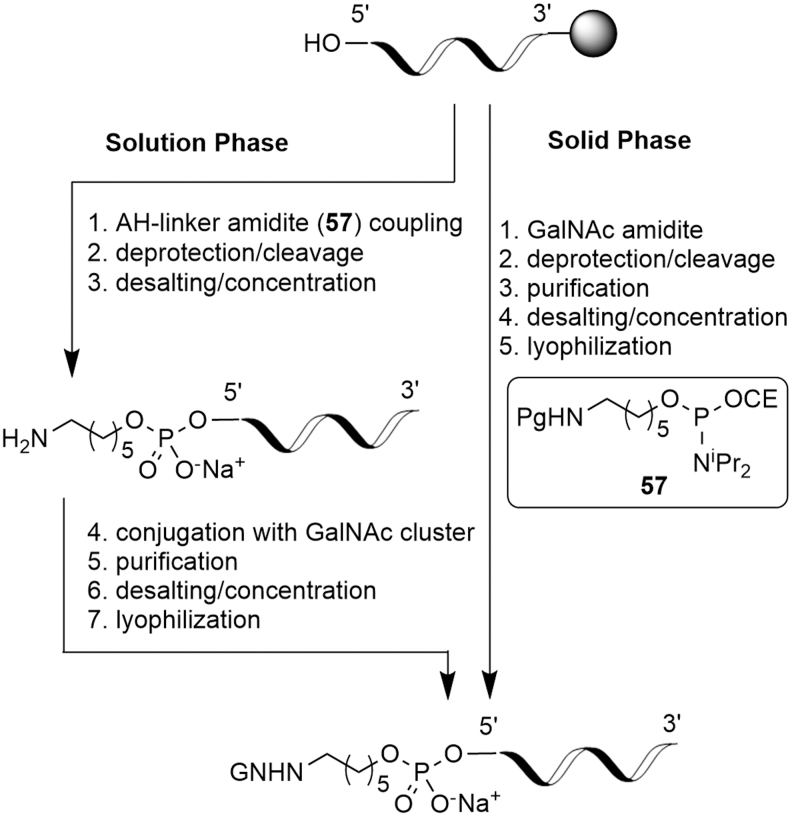
5′-GalNAc conjugation options.

Attachment of GalNAc at the 5′-oligonucleotide terminus serves as a useful example of the treatment of conjugate fragments. The conjugation can be done after the solid-phase synthesis or starting with the oligonucleotide construct loaded on the solid support (Fig. 20) [50].

As an example of the approach, this publication will focus on post-oligonucleotide synthesis conjugation, that is, “5′-GalNAc,” specifically bis-lysine cluster **58**. “GalNAc on oligonucleotide” and “3′-GalNAc” [41] will not be discussed, although the principles set out are equally applicable.

For 5′-GalNAc, completion of the oligonucleotide fragment synthesis is typically followed by the solid-phase coupling of a spacer amidite (such as **57**) and, finally, by the solution-phase coupling of the fully assembled GalNAc cluster, for example, **58**. Compared to naked oligonucleotides, GalNAc-conjugated oligonucleotides therefore contain two additional significant structural elements of the API. In a similar way to the (deoxy)ribose amidites, this fulfills the most fundamental criterion for an SM.

Based on ICH Q11, “enough of the drug substance manufacturing process should be described in the application. …” In the case of 5′-GalNAc conjugates, the 5′-GalNAc cluster is typically introduced in the final synthetic step offering fewer transformations than would be considered acceptable in the realm of traditional small molecules. However, the extensive downstream processing, including chromatographic purification, desalting by ultrafiltration/diafiltration, and isolation, may compensate for the reduced number of chemical transformations.

**FIG. 21.**
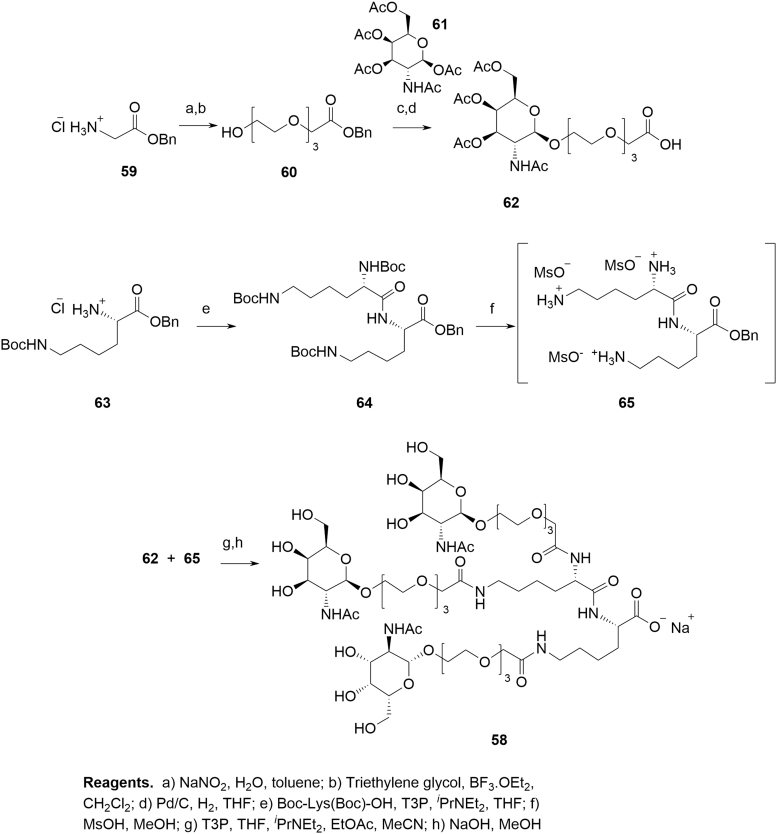
Synthesis of GalNAc Cluster **58**.

The linker used in 5′-GalNAc conjugates is typically a protected 6-aminohexyl phosphoramidite (6-AH) such as **57**. This is of a similar size to conventional small molecule SMs and can therefore be treated as such; it will not be discussed in any further detail. GalNAc cluster **58** is a typical example of its type and features a bis-lysine moiety to allow for sufficient branching and a triethyleneglycol spacer to enable additional separation of GalNAc from oligonucleotide (Fig. 21).

The convergent synthesis commences with benzyl glycinate **59**, which is converted to triethyleneglycol spacer **60** in a diazotization-promoted substitution [51]. Glycosidation with *N*-acetyl-d-galactosamine tetraacetate **61** mediated by TMSOTf provides β-anomer **62**. The *bis*-lysine coupling partner **65** is readily prepared from Boc-protected l-lysine benzyl ester **63**, which is coupled to bis-Boc-protected l-lysine under standard conditions (T3P, DIPEA) to give **64** followed by amine deprotection under acidic conditions to give **65**.

Crude **65** is used without further purification and coupled with **62** using T3P to give the protected GalNAc cluster, which is taken to the global deprotection step (aq. NaOH and MeOH) without further purification. GalNAc cluster **58** is purified using preparative HPLC.

**58** is an isolated, well-characterized, and stable amorphous material manufactured using a reliable and robust process. Its precursors are naturally occurring amino acid or sugar building blocks, which are readily available in ton scale in enantiomerically pure form. To date, the synthesis was carried out on multi-kg scale with appropriate analytical controls using standard techniques (HPLC).

It is important to point out preparative HPLC purification of **58** should not be considered a specific “unit operation” in the sense laid out in ICH Q11 Q&A. The choice of this technique is motivated rather by the lack of crystallinity of **58** than by failure of other purification techniques to provide material of sufficient quality.

**FIG. 22.**
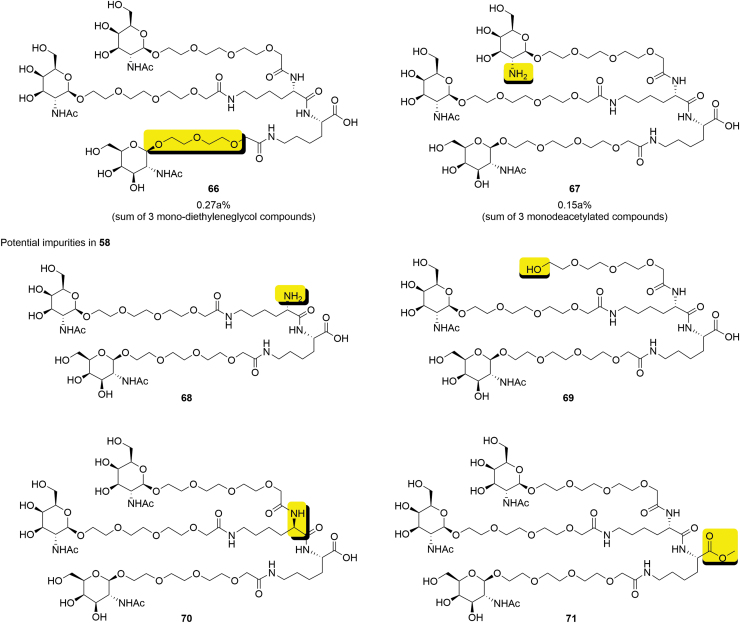
Impurities in GalNAc cluster **58** (impurity motif highlighted).

Even at a relatively early stage of development, a number of potential and actual impurities in **58** have been identified (Fig. 22). Potential process-related impurities will be continuously evaluated during further development. Any new unknown impurities detected above the reporting limit of the analytical method in future batches will be characterized, and their fate will be investigated in the subsequent processing steps, if needed.

The only impurities present in **58** at a level >0.10% area are critical impurities **66** (0.27% area^‡^) and **67** (0.15% area^§^). An impact of these two impurities on a drug substance (DS) CQA can be excluded based on their specification level in **58** and hence, no additional fate-of-impurity data are required. Impurities **68** and **69** are controlled by the process and their levels are below the reporting limit in **58**. It is advisable to ensure that analytical methods can assess the configuration of stereocenters, which are synthetically derived and/or prone to epimerization (eg, anomeric center of galactosamine or α-position of lysine amino acids in **70**). The nonreactive, noncritical impurity **71** has been shown to be depleted in the downstream purification step. In the context of ICH Q11, “impact on DS quality” is defined as level above the identification threshold. Since the amount present in GalNAc is significantly lower than the ID threshold in the API, an impact can be excluded.

The specification of **58** is based on current knowledge and will be revised as additional batch history data and/or process development data become available before the manufacture of the commercial drug substance batches.

Given the breadth of different GalNAc clusters used across the industry and the above-mentioned lack of regulatory guidance for oligonucleotides, a general recommendation regarding their acceptability as SM is difficult. Sponsors are encouraged to evaluate their GalNAc cluster using a science-based approach founded in the principles set forth in ICH Q11 and provide data demonstrating safety to patients.** The holistic approach outlined in ICH Q11 and associated Q&A provide a good framework for this assessment. Understanding of how the impurity profile of the SM affects the drug substance quality is necessary and should be supported by a sound specification for release testing of GalNAc. The application of GalNAc conjugates in a commercial setting is a very immature area; therefore, the advice presented illustrates scientific concepts that EPOC member companies feel are relevant to support GalNAc and related structures.

### Solid support

**FIG. 23.**
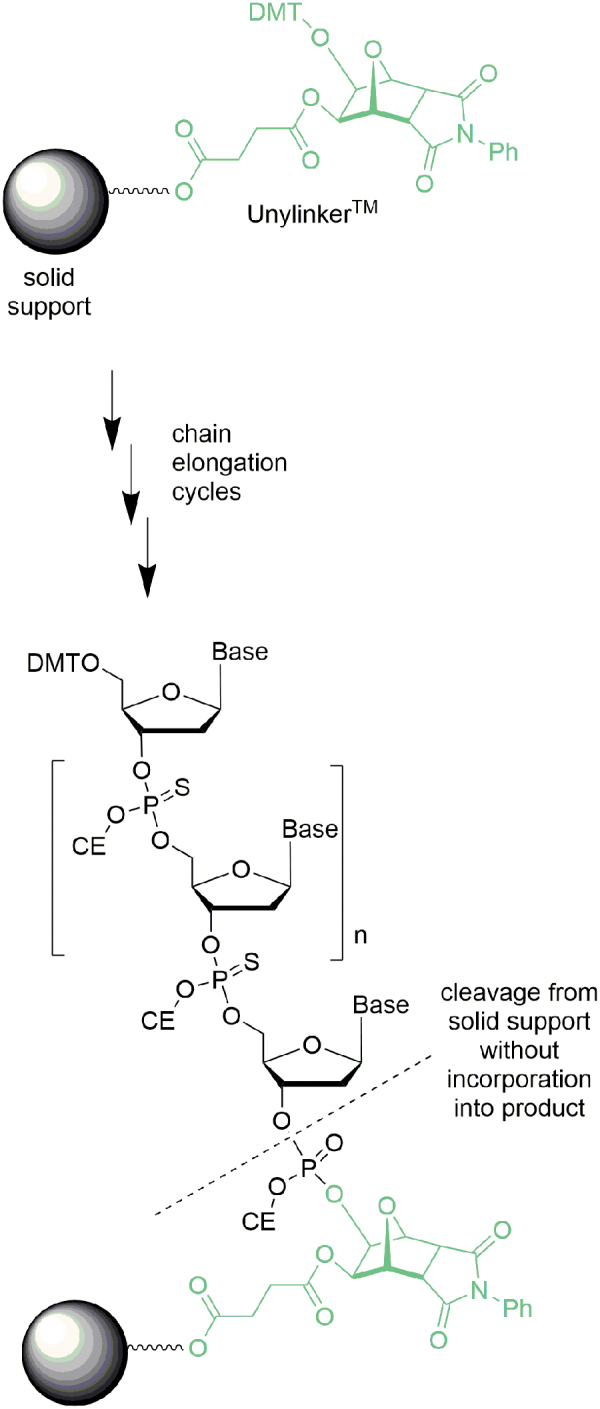
Relationship of NittoPhase UnyLinker^®^ and oligonucleotide.

One important element in most oligonucleotide syntheses is the synthesis support (eg, NittoPhaseHL^®^ and ^††^ Primer Support 5G^®^^‡‡^) and their derivatives functionalized with an appropriate linker (eg, UnyLinker*^®^*^§§^ and succinate). Typically, the first manufacturing step begins after deprotection of the commercial support (eg, NittoPhase UnyLinker HL350^®^) followed by coupling of the first nucleotide. Since the synthesis resin functioned as a stationary phase (or has even been described as a 3′-protecting group) for the automated synthesis, it contributed no material to the oligonucleotide at the end of the process and would be described as a noncontributory raw material (Fig. 23).

As with other noncontributory raw materials and reagents, the resin still exerts an effect on the synthesis and robust performance during oligonucleotide synthesis under GMP requires monitoring of appropriate attributes of the solid support. Sample preparation is key to examine impurities derived from solid supports and varies with the support [54], a few typical attributes are listed (Table 8).

**Table 8.** Standard Quality Attributes Generally Monitored for Solid Supports

Test	Method	Acceptance criterion
Appearance	Visual examination	Mixture of powder and aggregates
Identification	FTIR by ATR or NIR	Sample spectrum conforms to standard spectrum
DMT loading	Spectrophotometry	Measured in μmol/g
Impurities	HPLC	NMT 0.10% a/a

ATR, Attenuated total reflection spectroscopy; FTIR, Fourier transform infrared spectroscopy; NIR, near-infrared spectroscopy.

Although amidite coupling is generally very high yielding, there can be situations where the first coupling can be more challenging (eg, due to added steric hindrance/lower reactivity at the secondary alcohol of UnyLinker). Since oligonucleotide manufacturing is quite expensive, knowledge of the yield for the first coupling may be advantageous, but is not readily ascertained during manufacture because of the inability to sample the packed column. One approach to circumvent this difficulty is to apply preloaded supports where the first nucleotide or even 3′-GalNAc is already coupled to the resin. Such resins are available from most suppliers and loadings can be assayed, therefore increasing confidence for the subsequent manufacture.

In these cases, the input support contains structural elements of the final oligonucleotide and the noncontributory status cannot be applied. As a result, sponsors may seek to justify the preloaded support as an SM. It should be anticipated that loaded solid supports would require more extensive characterization than unmodified supports. For instance, performance characteristics of the support may involve derivatization and/or wet chemical techniques to examine loading capacity or impurities derived from the element that is bound to the loaded support (Table 9). There are complexities involved in the validation and routine use of these types of tests; so their necessity should be informed by a well-developed risk assessment that examines the capability of the assays and their ability to control for critical properties of the loaded solid support.

**Table 9.** Modified Solid Support Control and Characterization

Physical characteristics	Loading capacity	Quality attributes (cleavage may be required)
Particle size (microscopy)	Use test	Identity of loaded support (IR) or loaded entity (HPLC)
Swell volume (eg, in MeCN)	Use test	Related substances (HPLC)
Bulk density	Testing for stoichiometry	Water (KF)
		Residual solvents (GC)
		Loss on drying

IR, infrared.

## Conclusions

In this white paper, we provide guidance on the application of risk-based strategies, founded on the collective experience of member companies of the European Pharma Oligonucleotide Consortium. The purpose is to enable a more uniform approach to the justification of various general classes of oligonucleotide SMs.

As we describe, oligonucleotides are explicitly out of scope with respect to ICH Q11. EPOC member companies have, however, sought to apply the ethos and principles of ICH Q11 in their development activities, but in conjunction with awareness of the practices and knowledge of oligonucleotide processing. We recognize not only that step count in its traditional sense is not a helpful construct for SM justification during oligonucleotide processing but also that chromatographic purification and other downstream operations should serve to provide some mitigation. We describe how a detailed understanding of the synthetic steps in both SM and oligonucleotide processing can support a regulatory SM proposal addressing the importance and fate of impurities and providing confidence for patient safety.

We introduced our position using deoxyamidites **1–4** as the simplest and most common of the SMs used in oligonucleotide API manufacture. These clearly satisfy the criteria for SMs, in line with the guiding principles of ICH Q11:

- Impurity classes in these materials are well characterized and well understood
- The criticality of the various impurity classes toward impact on API quality are well understood (reactive, critical; reactive, noncritical; and nonreactive, noncritical)
- A high level of control is achievable (enabling very stringent purity-focused specifications)

For the related ribonucleoside amidite building blocks, such as the MOE amidites **23–26** and cEt amidites such as **42**, similar arguments can be applied. Although these materials are of greater synthetic complexity and can contain larger numbers of impurities than deoxyamidites, the control strategies employed and acceptance criteria in specifications are comparable and driven by the same risk-based principles. Analogously, with the appropriate understanding and control of impurities, amidite dimers or blockmers such as **56** would follow the ICH Q7 and Q11 SM principles as part of convergent oligonucleotide syntheses.

To exemplify oligonucleotide conjugates, a 5′-GalNAc case study of a linker-modified oligonucleotide was used to illustrate two further SM types to consider—the linker amidite **57** and the GalNAc cluster **58**. The GalNAc clusters were identified as a larger challenge; consequently, a general recommendation of their acceptability as SM is less straightforward; however, we recommend that sponsors apply the same risk- and science-based approaches defined in ICH Q11 to assess their own particular situation.

This article also gives consideration to the solid supports themselves. For the standard resins and the linkers used for attachment of the initial nucleotide unit to the resin, these are clearly defined as noncontributory raw materials, and the guidance around key attributes associated with use of these noncontributory raw materials is reiterated. In specific cases where a sponsor may choose to employ a functionalized solid support where a significant contributory element of the API is already present on the support, it is possible that further justification as an SM may be required, on a case-by-case basis, with a focus on the characterization and performance characteristics of the functionalized support.

In general, through the cross-industry examples provided in this article, we outline a clear and uniform approach to the designation of SM status for the amidite building blocks utilized in oligonucleotide API manufacture, firmly rooted in the principles of ICH Q11. We have also provided insight and guidance on the designation of more complicated SMs such as those required for oligonucleotide conjugates, as well as setting out a clear position relating to the solid supports required for synthesis. We believe that publication of these arguments will be of great value in enabling both sponsors and regulatory bodies to achieve greater clarity and harmony in justifying the status of SMs for use in cGMP oligonucleotide manufacture.

## Notes

^*^ EPOC article in preparation.

^†^ Discussions with deoxyamidite vendors indicate that typical activators applied include 4,5-dicyanoimidazole (DCI), 1*H*-tetrazole, 5-(ethylthio)-1*H*-tetrazole (ETT), 5-(benzylthio)-1*H*-tetrazole (BTT), 5-(3,5-bis(trifluoromethylphenyl)-1*H*-tetrazole), 5-(4-nitrophenyl)-1*H*-tetrazole, benzimidazolium triflate, and *N*-methylimidazolium triflate. Activators must possess an aqueous pKa value comparable to acetic acid [pKa_(aq)_ = 4.5] and produce a conjugate base that is both a good nucleophile and leaving group. The choice of activator used to phosphitylate the nucleoside precursor does not appear to impact amidite CQAs.

^‡^ Following oligonucleotide impurity conventions, this level accounts for three different impurities, which have a single diethylene glycol unit in one of the three spacer units.

^§^ This level accounts for three different impurities where any one of the three acetyl groups was cleaved.

^*^ *It is important to understand that the GalNAc cluster is a targeting moiety, which is cleaved from the active compound during metabolism and does not modulate the target. Toxicological coverage of the GalNAc cluster (including related impurities) is achieved through data generated with the API in animals and humans [52,53].

^††^ NittoPhase is a trademark of Nitto Denko Corporation.

^‡‡^ Primer 5G is a trademark of General Electric companies.

^§§^ UnyLinker is a trademark of Ionis Pharmaceuticals, Inc.
